# Microneedle-Array-Mediated Transdermal Delivery of GCV-Functionalized Zeolitic Imidazolate Framework-8 Nanoparticles for KSHV Treatment

**DOI:** 10.3390/ijms252312946

**Published:** 2024-12-02

**Authors:** Chengjing Liu, Xiuyuan Yin, Huiling Xu, Jianyu Xu, Mengru Gong, Zhenzhong Li, Qianhe Xu, Dongdong Cao, Dongmei Li

**Affiliations:** 1 Key Laboratory of Xinjiang Endemic and Ethnic Diseases, NHC Key Laboratory of Prevention and Treatment of Central Asia High Incidence Diseases, School of Medicine, Shihezi University, Shihezi 832003, China; 20211014607@stu.shzu.edu.cn (C.L.); xhl_hlx@126.com (H.X.); 20221014058@stu.shzu.edu.cn (J.X.); 20221014089@stu.shzu.edu.cn (M.G.); 20221014108@stu.shzu.edu.cn (Z.L.); caodongd@shzu.edu.cn (D.C.); 2 Shanghai Key Laboratory of Functional Materials Chemistry, Key Laboratory for Advanced Materials, School of Chemistry and Molecular Engineering, East China University of Science and Technology, Shanghai 200237, China; y30220302@mail.ecust.edu.cn (X.Y.); y10220019@mail.ecust.edu.cn (Q.X.)

**Keywords:** Kaposi’s sarcoma-associated herpesvirus, ganciclovir, microneedle, zeolite imidazolate framework-8, nano-delivery

## Abstract

Kaposi’s sarcoma-associated herpesvirus (KSHV) is a variety of the human gamma-herpesvirus that often leads to the occurrence of malignant tumors. In addition, the occurrence of Kaposi’s sarcoma is a major cause of death among AIDS patients. Ganciclovir (GCV) is the most widely used drug against KSHV infection in the clinic. GCV can restrict the in vivo synthesis of DNA polymerase in KSHV, thereby inhibiting the replication of the herpesvirus. However, GCV still suffers from poor specificity and transmembrane capabilities, leading to many toxic side effects. Therefore, developing a drug delivery system that increases GCV concentrations in target cells remains a significant clinical challenge. In this study, zeolite imidazole salt framework-8 (ZIF-8), a biocompatible porous material constructed by coordinating zinc ions and 2-methylimidazole, was used to load GCV. A nano-delivery system with a microneedle structure was also constructed using a polydimethylsiloxane (PDMS) microneedle mold to fabricate MN/GCV@ZIF-8 arrays. These arrays not only offered good skin-piercing capabilities but also significantly inhibited the cleavage and replication of the virus in vivo, exerting an anti-KSHV function. For these reasons, the arrays were able penetrate the skin’s stratum corneum at the tumor site to deliver GCV and play an anti-KSHV role.

## 1. Introduction

Kaposi’s sarcoma-associated herpesvirus (KSHV), also known as human herpesvirus 8 (HHV-8), belongs to the γ family of herpesvirus and can cause many lymphoproliferative malignancies, such as Kaposi’s sarcoma (KS), primary effusion lymphoma (PEL), and multicentric Castleman disease [1,2]. Today, the clinical treatment methods for KSHV-related malignancies mainly include surgery, radiation therapy, chemotherapy, and antiviral drug therapy [3]. However, surgery faces problems of postoperative tumor recurrence and metastasis, which can seriously affect quality of life and mortality among patients [4]. Moreover, simple surgical treatment cannot achieve satisfactory results. Although radiation therapy can shrink the tumor or make it disappear, such treatment can sometimes damage the surrounding tissue and lead to nausea and vomiting [5]. Due to their lack of targeting, chemotherapy drugs also have significant toxic side effects on normal cells [6]. Furthermore, because radiation and chemotherapy do not treat the underlying cause of the tumor, which is a viral infection, the rate of tumor recurrence is high.

Drug therapies for KSHV-related malignancies mainly use medications with antiviral effects to inhibit the expression of KSHV-related genes, thereby inhibiting tumor growth [7]. However, some antiviral drugs have low transmembrane capabilities with no tumor-targeting characteristics, and they often need continuous administration. This extended use can produce systemic toxic side effects, which have serious physiological and psychological impacts on patients. Therefore, researchers are currently exploring methods to avoid the toxic side effects of drugs and efficiently treat cancer. In recent years, the continuous development of nanotechnology and its interactions with multiple disciplines have introduced new ideas for the treatment of cancer. Nano-drug delivery systems (NDDSs) constructed using different types of nanocarriers have been developed by researchers to overcome the aforementioned problems in the field of cancer treatment [8].

Ganciclovir (GCV) is a 2-deoxyguanine nucleotide analog that inhibits the replication of herpesviruses in vivo, including herpes simplex virus, varicella–zoster virus, Epstein–Barr virus, and cytomegalovirus [9]. After entering virus-infected cells, GCV is first phosphorylated to monophosphate by the protein kinase homolog of the virus and then activated into triphosphates by cell kinases. Triphosphates can inhibit viral replication by competing to inhibit viral DNA polymerase and binding to viral DNA chains, thereby disrupting DNA chain elongation [10,11]. GCV can inhibit the DNA polymerase of KSHV. However, the water solubility of this antiviral gives GCV poor transmembrane capabilities against the plasma membranes of lipid-soluble cells and low specificity against the site of infection. Moreover, the oral bioavailability of GCV is only about 5%. Long-term high-dose treatment leads to many toxic side effects, such as thrombocytopenia, persistent myelosuppression, and severe liver and kidney damage [12]. Therefore, there is an urgent need to develop a drug delivery system that effectively delivers GCV.

Nanotechnology is expected to solve the poor transmembrane capabilities and targeting issues of ganciclovir. Zeolite imidazole salt framework-8 (ZIF-8) is a biocompatible porous material constructed by coordinating zinc ions with 2-methylimidazole [13,14]. This material’s simple preparation process, large specific surface area [14,15], good chemical stability, and unique pH response decomposition characteristics make ZIF-8 a promising drug delivery vehicle for effective GCV delivery against the mildly acidic environment at the site of infection.

On the other hand, microneedle drug delivery has been widely used in the biomedical field [16,17]. Microneedle drug delivery is a micro-invasive transdermal drug delivery method that combines the advantages of using subcutaneous injections and skin patches [18,19]. The microneedle penetrates the cuticle of the epidermis of the skin and forms tiny channels along which the drug enters the skin, increasing the drug’s permeability and accumulation at specific sites. However, soluble microneedles obtained using traditional preparation methods cannot be fully and effectively utilized after entering the skin. Because the skin has a certain elasticity, the microneedles cannot be completely submerged, which limits the efficiency of drug delivery and leads to waste.

To address these challenges, we designed a soluble microneedle patch for GCV delivery. GCV was loaded into ZIF-8, and the prepared tip was shaped like a torch. GCV@ZIF-8 was enriched at the tip of the microneedle. The torch microneedle increased the contact area and drug loading, thereby achieving rapid drug release from the microneedle and local drug administration in the treatment of KSHV. Moreover, the torch microneedle geometry includes a sharp skin penetration tip, a wide skin-interlocking body, and a narrow base to create mechanical support on a flexible hydrocolloid patch. These features increase the accuracy of skin penetration into irregular surfaces and further improve drug utilization. Overall, using a soluble microneedle body reduces the waste caused by the inability of the drug to fully enter the skin.

In this study, the combination of GCV@ZIF-8 and microneedle arrays achieved excellent GCV delivery, showing that this novel composite nanomaterial may provide a promising alternative for the treatment of KSHV infection.

## 2. Results and Discussion

### 2.1. Preparation and Characterization of GCV@ZIF-8

**Scheme 1:**
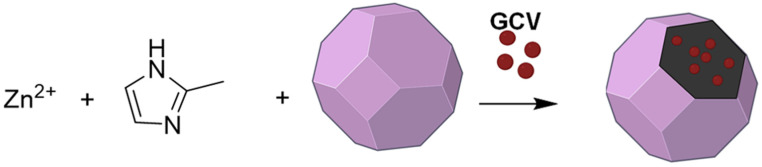
The synthesis method of GCV@ZIF-8.

**Figure 1:**
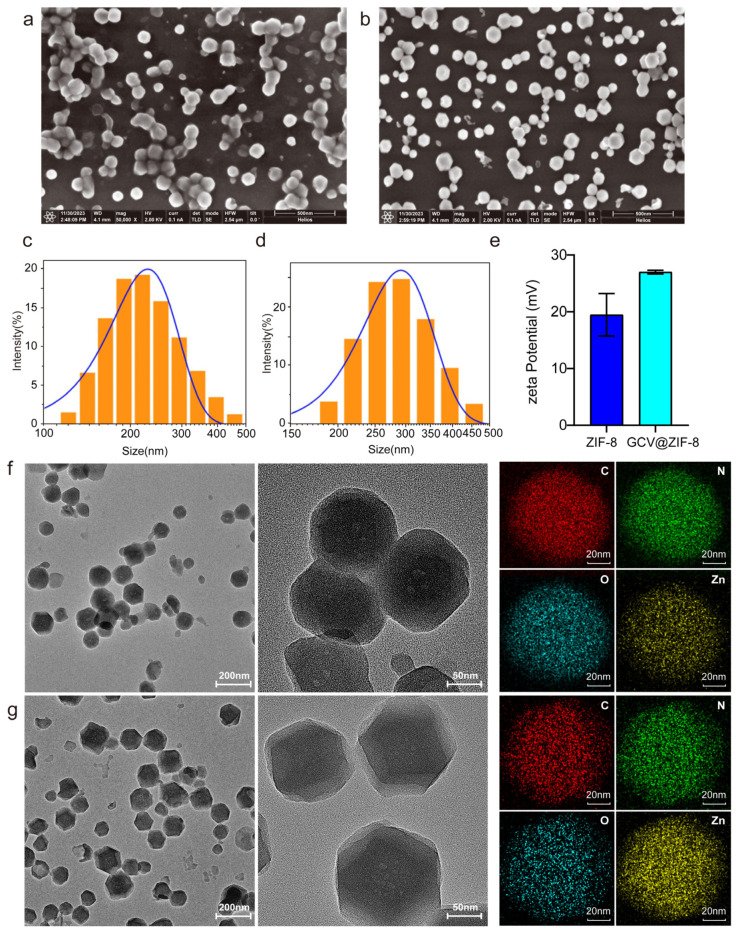
Synthesis and characterization of ZIF-8 and GCV@ZIF-8 NPs: (**a**,**b**) SEM images of ZIF-8 and GCV@ZIF-8 NPs; scale bar: 500 nm. (**c**,**d**) Particle size distribution of ZIF-8 and GCV@ZIF-8 NPs measured with DLS. (**e**) Zeta potential of ZIF-8 and GCV@ZIF-8 NPs measured with DLS. (**f**) TEM images of ZIF-8 and corresponding elemental mapping images of C, N, O, and Zn. (**g**) TEM images of GCV@ZIF-8 and corresponding elemental mapping images of C, N, O, and Zn.

Zinc-based zeolite imidazole salt framework-8 (ZIF-8) is composed of tetrahedral zinc ions and imidazolic acid ligands with covalent bonds. ZIF-8 features a high surface area and porosity [20]. The drug-carrying nano-complex GCV@ZIF-8 was prepared as shown in Scheme 1. GCV was loaded into ZIF-8 via in situ encapsulation, and the morphologies of ZIF-8 and GCV@ZIF-8 were observed using SEM (Figure 1a,b). At the same time, we tested the particle sizes of ZIF-8 and GCV@ZIF-8. The particle size of ZIF-8 was about 220 nm (Figure 1c). After GCV loading, the particle size slightly increased to about 300 nm (Figure 1d). Further detection showed that the potential of ZIF-8 was 19.47 ± 3.74, while that of GCV@ZIF-8 was 27.0 ± 0.34 (Figure 1e). The morphologies of ZIF-8 (see Figure 1f) and GCV@ZIF-8 (see Figure 1g) were observed via TEM. ZIF-8 and GCV@ZIF-8 exhibited dodecahedral structures, and TEM element maps were used to visualize the spatial distribution of the elements C, N, O, and Zn in ZIF-8 and GCV@ZIF-8.

### 2.2. pH Responsiveness and Cytotoxicity of GCV@ZIF-8

**Figure 2:**
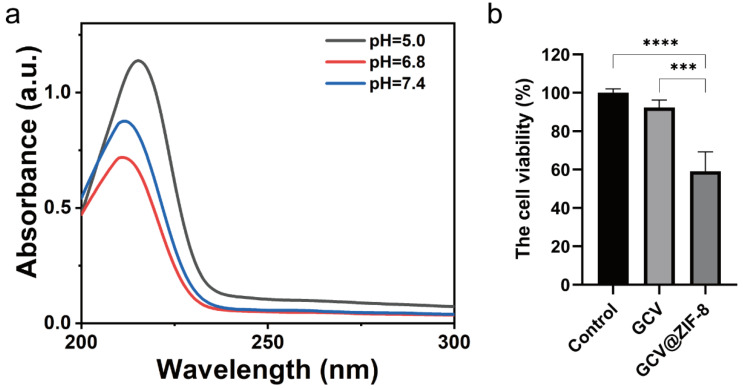
The (**a**) pH responsiveness and (**b**) cell toxicity of GCV@ZIF-8; data are presented as the mean ± SD for three independent experiments; *** *p* < 0.001; **** *p* < 0.0001.

Under neutral conditions, ZIF-8 maintains structural stability, while under slightly acidic conditions it decomposes and releases drugs [21,22]. This pH response has led to ZIF-8 being widely studied as a drug delivery carrier. To verify the pH response capability of GCV@ZIF-8 NPs, the UV absorption of GCV was measured by dissolving the same amount of material in different pH buffer solutions. We found that the release of GCV was more obvious in a buffer solution with pH 5.0 (Figure 2a). Therefore, GCV@ZIF-8 is a responsive drug delivery system. After the acid-sensitive group breaks apart, it can achieve targeted delivery to a specific site.

To further investigate the toxicity of GCV@ZIF-8 NPs on KSHV-positive cells, we established a free-GCV group for the GCV@ZIF-8 group to detect the cells’ capabilities with an MTT assay (Figure 2b, Supplementary Table S1). We found that free GCV itself has low transmembrane capacity and has difficulties entering KMM cells to resist KSHV (*p* < 0.001). Additionally, the GCV@ZIF-8 group led to a significant decrease, indicating that GCV@ZIF-8 offers better anti-tumor effects.

### 2.3. Preparation and Characterization of MN/GCV@ZIF-8

**Figure 3:**
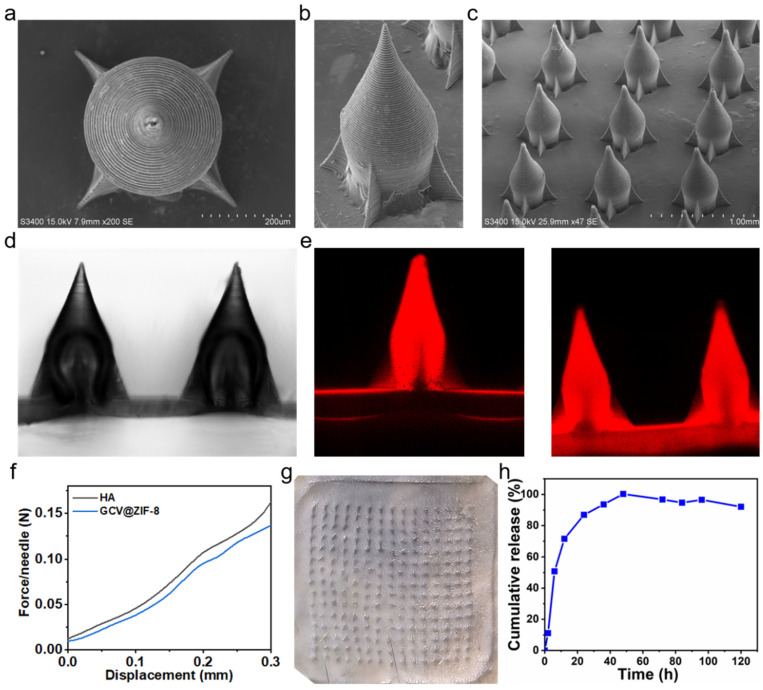
Characterization and SEM images of the MNs: (**a**) overhead view of MN/DOX + GCV; (**b**) high-magnification views of MNs; (**c**) lateral views of MNs; (**d**) image of microneedles taken with an inverted microscope; (**e**) confocal images of the microneedle; (**f**) stress curves of microneedles; (**g**) the appearance of the skin after treatment with MNs; (**h**) the penetration of drug content into the skin after inserting MNs at different timepoints.

Here, we doped GCV@ZIF-8 into HA and prepared nanomaterial-supported soluble microneedles (MN/GCV@ZIF-8) with a PDMS mold for drug delivery in vivo. The needle body matrix and backing layer materials were composed of HA with different molecular weights, thus taking advantage of the biocompatibility and degradability of HA. Scanning electron microscopy (SEM) was used to detect the top, side, and magnification morphology of the microneedles (Figure 3a–c). The entire structure consisted of a 16.5 × 16.5 mm patch and a 15 × 15 needle array with 0.31 mm spacing and a height of 800 μm. The torch height was 512 μm, the pyramid base height was 288 μm, and the base width was 400 μm. The needle body was observed using inverted and confocal microscopes (Figure 3d,e).

### 2.4. Mechanical Properties of MN/GCV@ZIF-8

The stratum corneum of the skin prevents the penetration of most drugs, resulting in poor drug absorption [23]. To investigate whether the MN/GCV@ZIF-8 arrays could penetrate back skin under pressure, we tested the actual skin penetration capabilities of the arrays loaded with Rhodamine using fresh rat back skin. The mechanical strength of MN/GCV@ZIF-8 was thus determined (Figure 3f). When the displacement was 300 μm, the fracture force of a single needle was about 0.08 N. These mechanical strengths are sufficient to meet the minimum force required to penetrate human skin (0.058 N), ensuring that MN/GCV@ZIF-8 can be successfully inserted into the skin without breaking (Figure 3g).

### 2.5. Drug Release Study

The dissolution behavior of the matrix in a microneedle array directly affects drug release [24,25]. To further investigate the dissolution properties of MN/GCV@ZIF-8 in vitro, the release of RhB@GCV@ZIF-8 at 0 h, 1 h, 2 h, 4 h, 6 h, 12 h, 18 h, 24 h, 36 h, 48 h, and 72 h was tested using a Franz diffusion cell. The release curve showed that the soluble microneedles continuously released drugs. The release reached 90% in 24 h, and the release level tended to be stable and remained above 90% after continuous monitoring (Figure 3f), indicating that the release was complete after 24 h.

### 2.6. Skin Penetration Studies

**Figure 4:**
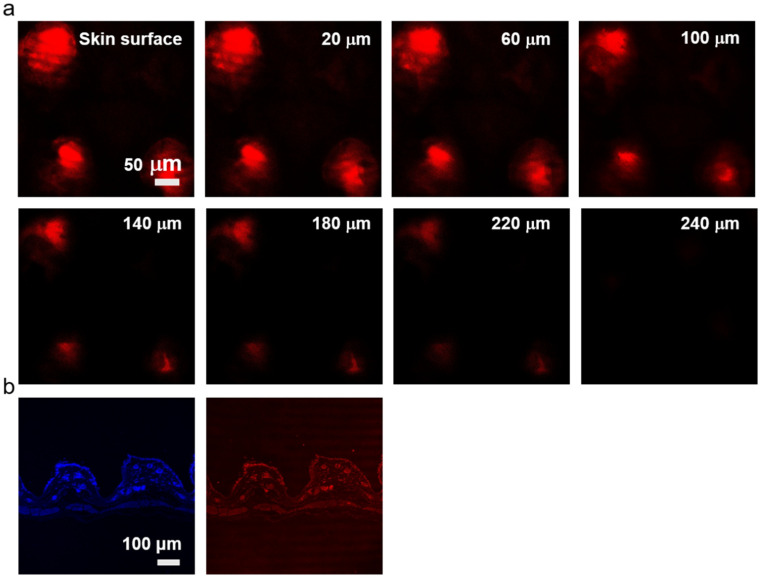
Confocal images of the microneedles: (**a**) the appearance of the skin after treatment with MNs; (**b**) confocal laser tomography images of DAPI (Blue) and Rhodamine (Red) staining with microneedles.

To further determine the prudence of MN/GCV@ZIF-8 insertion, the skin was scanned using a confocal laser scanning microscope (CLSM). As shown in Figure 4a, the fluorescence signal of Rhodamine B was strongest on the skin surface and gradually weakened with an increase in the microneedle insertion depth. Even at a depth of 220 μm, a weak fluorescence signal could still be detected, indicating that, after the initial dissolution of the microneedles, the GCV drug reached a depth of 220 μm, ensuring highly efficient GCV delivery. In addition, after microneedle administration, the red fluorescence of Rhodamine B and the channel formed by the microneedles could be clearly observed on the skin of the mice. The channel was 280 μm in size (Figure 4b)—large enough to form a microneedle dermal drug delivery system and achieve the desired therapeutic effect.

### 2.7. Anti-Tumor Effects In Vivo

**Figure 5:**
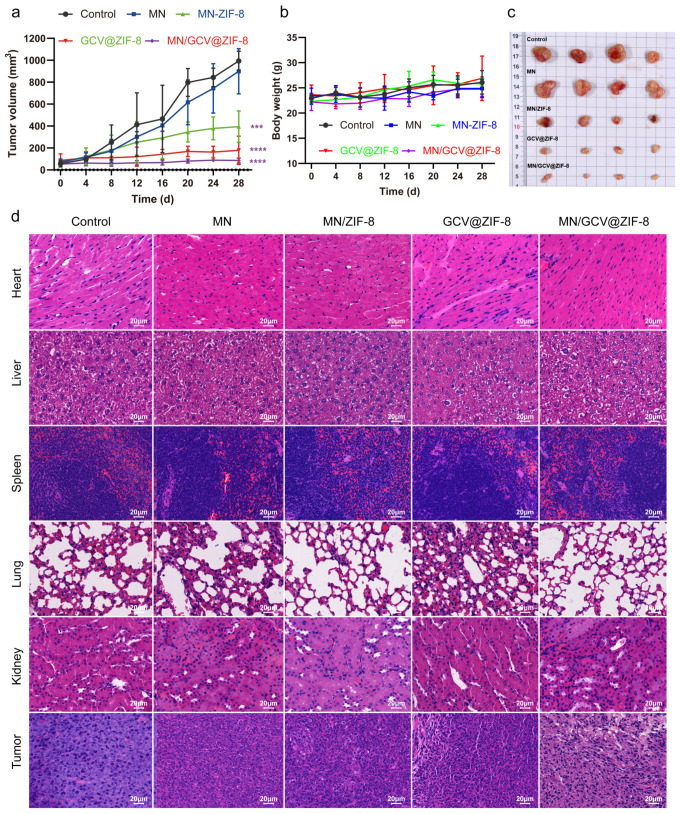
The anti-tumor effects of MNs in vivo: (**a**) tumor volume of each group; (**b**) body weight records of nude mice; (**c**) tumors isolated from nude mice; (**d**) HE staining of different organs (heart, liver, spleen, lungs, kidneys, and tumor) in nude mice. Data are presented as the mean ± SD for three independent experiments; *** *p* < 0.001; **** *p* < 0.0001.

In order to further verify the anti-tumor function of MN/GCV@ZIF-8, we further evaluated its therapeutic effect in vivo by constructing a tumor model induced by KSHV infection in nude mice. The curve of the tumor volume over time was recorded. It was shown that, compared with the control group, all treatment groups had different degrees of tumor inhibition within 28 days, where the blank MN group showed weaker inhibition of tumor growth, while the MN/GCV@ZIF-8 group showed the best inhibition of tumor growth (Figure 5a, Supplementary Table S2, *p* < 0.0001). At the same time, it was found that the weight of the nude mice in each group did not decrease significantly (Figure 5b, *p* > 0.05). Tumor tissue images of nude mice in each group after 28 days of treatment showed the anti-tumor effect of the microneedles (Figure 5c), which was consistent with tumor volume. In addition, after the experiment, major organs of the nude mice were collected for tissue sections and H&E staining, and no obvious physiological abnormalities were found in any of the groups (Figure 5d), which also proves that MN and MN/GCV@ZIF-8 have good biocompatibility.

### 2.8. Inhibitory Effects of MNs on the Expression Levels of KSHV Genes

**Figure 6:**
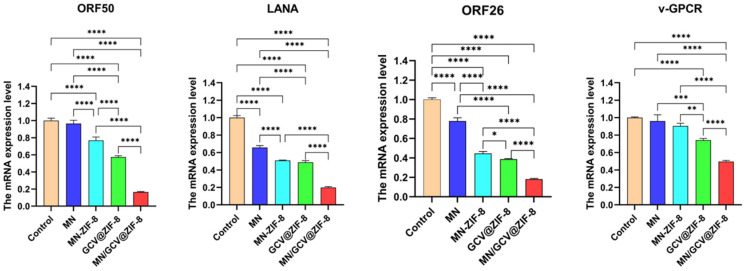
KSHV gene expression levels in the tumor. Data are presented as the mean ± SD for three independent experiments; * *p* < 0.05; ** *p* < 0.01; *** *p* < 0.001; **** *p* < 0.0001.

After KSHV infection, the virus can appear in one of two states: latent or lytic [26]. In the latent state, the virus expresses only limited proteins to avoid the host’s immune response and protect the survival of the virus. The lytic viral genome evolves into linear DNA, which facilitates large-scale viral replication [27,28]. Therefore, we further analyzed the expression levels of the latent KSHV gene *LANA* and the lytic KSHV genes *ORF50*, *ORF26*, and *v-GPCR* in tumors. We found that the results were consistent with the tumor size, and the MN/GCV@ZIF-8 group could significantly inhibit the expression of KSHV genes and exert antiviral and anti-tumor effects (Figure 6, Supplementary Table S3, *p* < 0.05).

Based on the above research, it can be seen that the nano-complex combined with microneedle transdermal drug delivery can enhance drug solubility, permeability, stability, and targeting. It will have broad development prospects in the delivery of biotechnology drugs. However, there are also many challenges. The production process and quality control standards of microneedles have not been fully standardized, including the size, shape, mechanical strength, drug content, application time, safety of microneedles, etc. This has led to some resistance to the application and promotion of microneedles. In addition, there may be various risks and challenges in the use process. If the microneedle is used incorrectly, the penetration depth of the microneedle will vary, resulting in an inaccurate delivery of the drug dose. Whether there is long-term cumulative toxicity of microneedles needs further study. Some special diseases can also affect the skin properties of patients, and the dosage and size of microneedles should be taken into account. This all needs to be fully evaluated and addressed before entering clinical use. We believe that, with further efforts, the above problems will be gradually solved. Microneedles will show their unique advantages and may replace the traditional drug delivery method to become a new mainstream form of drug delivery.

## 3. Materials and Methods

### 3.1. Materials

Zinc nitrate hexahydrate (Zn(NO_3_)_2_·6H_2_O) and 2-methylimidazole (2-MIM) were obtained from Aladdin. Trypan blue was purchased from Macklin. GCV and RhB were purchased from Adamas-beta.

### 3.2. The Preparation and Characterization of GCV@ZIF-8

GCV (100 mg) was dissolved in 2 mL of DMSO for ultrasonic use. Then, 240 mg of Zn(NO_3_)_2_·6H_2_O was added to 10 mL of methanol and stirred at room temperature until completely dissolved. In total, 400 μL (20 mg) of the GCV preparation solution was next added to the mixture and stirred evenly. Then, 2-MIM (480 mg) was dissolved in 10 mL of methanol and added to the above solution. The reaction mixture was stirred at room temperature for 12 h, washed with 50% ethanol, and centrifuged 3 times. The centrifugation speed and time were 8000 rpm and 10 min, respectively. After drying at 40 °C for 12 h, the white solid product GCV@ZIF-8 was obtained.

We next ultrasonically combined the GCV@ZIF-8 mixture with 50 μg/mL ultrapure water. Then, scanning electron microscopy (SEM, SU8010, Hitachi, Tokyo, Japan) and transmission electron microscopy (TEM, HT7700, Tokyo, Japan) were used to characterize the morphology of the nanomaterials. Using S2-sized nanoparticles (Nano ZS90, London, UK), we measured the hydrodynamic diameter and zeta potential.

### 3.3. The pH Response Ability of GCV@ZIF-8

To verify the pH response ability of GCV@ZIF-8, the same amount of material was dissolved in PBS buffer solution with pH values of 5.5, 6.8, and 7.4. After ultrasound, the release of GCV at different pH values was determined via ultraviolet absorption at 200–300 nm.

### 3.4. Cell Culture and Toxicity Detection

KMM cells are KSHV-positive cells. The specific operational methods for cell culturing and subsequent cell experiments were mainly based on those used in previous work by our team. An MTT assay was used to detect cell toxicity. KMM cells were placed in 96-well plates at a density of 5000 cells per well and incubated overnight in a DMEM medium (Gibco) containing 10% FBS (BI) at 37 °C and 5% CO_2_. The control, GCV, and GCV@ZIF-8 cells were treated for 24 h, and 20 μL of MTT (Solarbio, Beijing, China) solution was added to each well for 4 h. Then, we added 100 μL of DMSO (Solarbio, Beijing, China) and measured the absorbance at 490 nm.

### 3.5. Preparation of Soluble Microneedles Loaded with Ganciclovir (MN/GCV@ZIF-8)

We dissolved 40 mg of GCV@ZIF-8 materials in 1 mL of ultrapure water to create a 40 mg/mL solution. After ultrasonic dissolution, we dissolved 50 mg of hyaluronic acid (HA, 40–100 kDa) in the solution (HA:GCV@ZIF-8:H_2_O = 50 mg:40 mg:1 mL) to produce a gel on the surface of the polydimethylsiloxane (PDMS) templates. The mixture was then centrifuged at 3950 rpm for 6 min at room temperature. Next, we removed the supernatant and dried it in an oven at 120 °C for 2 h to obtain the initial needle tip. The needle tip was prepared after repeatedly adding liquid and drying via centrifuge. In total, 10% HA (200–400 kDa) was added to the PDMS template, ventilated, and dried in the shade at room temperature for 24 h. Next, the 15 × 15 array of loaded self-locking micro-GCV nanoparticles was synthesized and named MN/GCV@ZIF-8.

### 3.6. Determining the Force Curve of MN/GCV@ZIF-8

Scissors were used to trim the flat edges of the MN/GCV@ZIF-8 strips after adhering the microneedles flat to the glass slide with a double-sided adhesive backing layer to ensure that each root tip of the testing sensor was evenly exposed to the relevant mechanics and stress. The microneedles were placed face up on the central test bench of the instrument, and then they were compressed at a constant rate (0.1 mm/min) using parallel metal plates, to a maximum force of 100 N. The speed was close to the tip of the needle; once the top of the needle tip was touched, the mechanical test began, and the pressure was sensed to obtain a curve of pressure changes with displacement.

### 3.7. Morphological Characterization of MN/GCV@ZIF-8

To increase the electrical conductivity of the surface, a thin layer of gold was applied to the microneedle array. The instrument operated at 15 kV and captured images at magnifications between 50 and 400×.

### 3.8. The Permeation of MN/GCV@ZIF-8 Determined In Vitro

We punctured fresh rat skin with microneedles containing the fluorescent dye Rhodamine B, with the microneedle tips all inserted, and pressed for 15 min. Then, the skin was fixed on microscope slides and viewed under a confocal laser scanning microscope (CLSM) under an excitation wavelength of 568 nm, with spacing of 20 microns on the x, y, and z axes, for vertical scanning and fluorescence image analysis.

### 3.9. MN/GCV@ZIF-8 Release Test

Next, we removed the hair and isolated the whole layer of skin on the backs of the Balb/c mice. We also removed the subcutaneous tissue and pressed the soluble MN/GCV@ZIF-8 patch containing Rhodamine into the skin. The dermal side of the skin was fixed in the transdermal diffusion pool facing the receiving pool. The receiving tank (20 mL of PBS, pH = 7.4) was maintained at 37 °C and stirred at 600 rpm. Then, 600 μL of the receiving solution was taken to measure the fluorescence intensity at 0 h, 1 h, 2 h, 4 h, 6 h, 12 h, 18 h, 24 h, 36 h, 48 h, and 72 h. Finally, the cumulative transdermal release of drugs in the MN/GCV@ZIF-8 patch was measured.

### 3.10. Animal Assay

The animal experiments were approved by the Ethics Committee of Shihezi University. Twenty-five female Balb/c nude mice of the same age were injected with 3 × 10^6^ KMM cells to construct the model. When the tumor volume was about 100 mm^3^, the mice were randomly divided into 5 groups, named the control group, blank microneedle group (MN), ZIF-8 microneedle group (MN/ZIF-8), GCV@ZIF-8 group, and MN/GCV@ZIF-8 group. The tumor weight and volume of the mice were monitored every four days. The control group received no treatment, while the MN and MN/GCV@ZIF-8 groups were treated every four days. The drug injection group was treated via peritumoral injection with 100 mL of ganciclovir nanomaterials. The MN/GCV@ZIF-8 group was anesthetized with isoflurane for pets, followed by affixing MN/GCV@ZIF-8 to the tumors of the mice and gently pressing it into the skin. After 28 days, the heart, liver, spleen, lungs, kidneys, and tumors were isolated after euthanatizing the mice, and then fixed in formalin for H&E section staining.

### 3.11. RT-PCR

We fully ground 20 mg of tumor tissue in 1 mL of pre-cooled TRIpure and extracted RNA using chloroform and isopropyl alcohol. The cDNA was synthesized with an EntiLink™ 1st Strand cDNA Synthesis Super Mix (ELK Biotechnology, Wuhan, China), followed by real-time PCR (QuantStudio 6 Flex System PCR, Life technologies, San Francisco, USA) to amplify the target fragment described in our previous study [29] and the primer, as shown in Table 1.

### 3.12. Statistical Analysis

The GraphPad Prism 9.0 software was used to analyze the experimental data, and *p* < 0.05 was considered statistically significant. The data were then expressed as the mean ± standard deviation.

## 4. Conclusions

In this study, we designed a delivery mode combining pH-responsive functionalized ZIF-8 nanoparticles and a microneedle array for treating KSHV infections. This mode offered the effective delivery of GCV@ZIF-8 nanoparticles to the site of the KSHV infection. Furthermore, the GCV@ZIF-8 nanoparticles decomposed under slightly acidic conditions, and GCV was released to play a key role in inhibiting the infection caused by KSHV. Overall, MN/GCV@ZIF-8 has good mechanical properties for piercing the skin to achieve subcutaneous drug release, with the depth of administration reaching up to 220 μm. Therefore, this MN/GCV@ZIF-8 array embedded with ZIF-8 nanoparticles could be an efficient candidate for delivering drugs to achieve an important anti-KSHV function.

## Figures and Tables

**Table 1:** The primer sequence.

Primer Name	Sequence (5′-3′)	
Actin	Sense	CGGAACCGCTCATTGCC
	Antisense	ACCCACATCGTGCCCATCTA
ORF26	Sense	CGAATCCAACGGATTTGACCTC
	Antisense	CCCATAAATGACACATTGGTGGTA
ORF50	Sense	GAGTCCGGCACACTGTACC
	Antisense	AAACTGCCTGGGAAGTTAACG
LANA	Sense	AGCCACCGGTAAAGTAGGAC
	Antisense	GATGTGACCTTGGCGATGAC
v-GPCR	Sense	GTGCCTTACACGTGGAACGTT
	Antisense	GGTGACCAATCCATTTCAAGA

## Data Availability

All experimental data within this article are available from the corresponding author upon reasonable request.

## Supplementary Materials

The following supporting information can be downloaded at https://www.mdpi.com/article/10.3390/ijms252312946/s1.
