# Supplementary material for: Microneedle-Array-Mediated Transdermal Delivery of GCV-Functionalized Zeolitic Imidazolate Framework-8 Nanoparticles for KSHV Treatment
Source: Int J Mol Sci. 2024 Dec 2;25(23):12946. doi: 10.3390/ijms252312946 (PMC11641177; doi:10.3390/ijms252312946)
Supplement: Supplementary file 1 [file ijms-25-12946-s001.zip › ijms-3309459-supplementary.pdf]

Supplementary Table S1 Cell toxicity:

Tukey's Multiple Comparisons Test	Mean Diff.	95.00% CI of Diff.	Below Threshold?	Summary	Adjusted P-Value	
Control vs. GCV	7.623	-3.905 to 19.15	No	ns	0.2034	A-B
Control vs. GCV@ZIF-8	40.83	28.38 to 53.28	Yes	****	<0.0001	A-C
GCV vs. GCV@ZIF-8	33.21	20.76 to 45.66	Yes	***	0.0002	B-C

Supplementary Table S2 Tumor Volume:

Tukey's Multiple Comparisons Test	Mean Diff.	95.00% CI of Diff.	Below Threshold?	Summary	Adjusted P-Value	
Control vs. MN	93.57	-196.0 to 383.1	No	ns	0.8523	A-B
Control vs. MN-ZIF-8	599.3	309.7 to 888.9	Yes	***	0.0001	A-C
Control vs. GCV@ZIF-8	815.2	525.7 to 1105	Yes	****	<0.0001	A-D
Control vs. MN/GCV@ZIF-8	908.3	618.7 to 1198	Yes	****	<0.0001	A-E
MN vs. MN-ZIF-8	505.7	216.2 to 795.3	Yes	***	0.0006	B-C
MN vs. GCV@ZIF-8	721.7	432.1 to 1011	Yes	****	<0.0001	B-D
MN vs. MN/GCV@ZIF-8	814.7	525.1 to 1104	Yes	****	<0.0001	B-E
MN-ZIF-8 vs. GCV@ZIF-8	216	-73.60 to 505.5	No	ns	0.1974	C-D
MN-ZIF-8 vs. MN/GCV@ZIF-8	309	19.43 to 598.6	Yes	*	0.0339	C-E
GCV@ZIF-8 vs. MN/GCV@ZIF-8	93.03	-196.5 to 382.6	No	ns	0.8548	D-E

Supplementary Table S3 KSHV gene expression analysis:

ORF 50						
Tukey's Multiple Comparisons Test	Mean Diff.	95.00% CI of Diff.	Below Threshold?	Summary	Adjusted P-Value	
Control vs. MN	0.03514	-0.04129 to 0.1116	No	ns	0.5772	A-B
Control vs. MN-ZIF-8	0.231	0.1546 to 0.3075	Yes	****	<0.0001	A-C
Control vs. GCV@ZIF-8	0.4252	0.3488 to 0.5017	Yes	****	<0.0001	A-D
Control vs. MN/GCV@ZIF-8	0.8355	0.7590 to 0.9119	Yes	****	<0.0001	A-E
MN vs. MN-ZIF-8	0.1959	0.1195 to 0.2723	Yes	****	<0.0001	B-C
MN vs. GCV@ZIF-8	0.3901	0.3137 to 0.4665	Yes	****	<0.0001	B-D
MN vs. MN/GCV@ZIF-8	0.8003	0.7239 to 0.8768	Yes	****	<0.0001	B-E
MN-ZIF-8 vs. GCV@ZIF-8	0.1942	0.1178 to 0.2706	Yes	****	<0.0001	C-D
MN-ZIF-8 vs. MN/GCV@ZIF-8	0.6044	0.5280 to 0.6809	Yes	****	<0.0001	C-E
GCV@ZIF-8 vs. MN/GCV@ZIF-8	0.4102	0.3338 to 0.4866	Yes	****	<0.0001	D-E
LANA						
Tukey's Multiple Comparisons Test	Mean Diff.	95.00% CI of Diff.	Below Threshold?	Summary	Adjusted P-Value	
Control vs. MN	0.3427	0.2956 to 0.3899	Yes	****	<0.0001	A-B
Control vs. MN-ZIF-8	0.4912	0.4440 to 0.5384	Yes	****	<0.0001	A-C
Control vs. GCV@ZIF-8	0.5129	0.4658 to 0.5601	Yes	****	<0.0001	A-D
Control vs. MN/GCV@ZIF-8	0.8011	0.7540 to 0.8483	Yes	****	<0.0001	A-E
MN vs. MN-ZIF-8	0.1485	0.1013 to 0.1956	Yes	****	<0.0001	B-C
MN vs. GCV@ZIF-8	0.1702	0.1230 to 0.2174	Yes	****	<0.0001	B-D
MN vs. MN/GCV@ZIF-8	0.4584	0.4112 to 0.5056	Yes	****	<0.0001	B-E

MN-ZIF-8 vs. GCV@ZIF-8	0.02173	-0.02544 to 0.06890	No	ns	0.5754	C-D
MN-ZIF-8 vs. MN/GCV@ZIF-8	0.3099	0.2628 to 0.3571	Yes	****	<0.0001	C-E
GCV@ZIF-8 vs. MN/GCV@ZIF-8	0.2882	0.2410 to 0.3354	Yes	****	<0.0001	D-E
ORF 26						
Tukey's Multiple Comparisons Test	Mean Diff.	95.00% CI of Diff.	Below Threshold?	Summary	Adjusted P-Value	
Control vs. MN	0.2218	0.1692 to 0.2743	Yes	****	<0.0001	A-B
Control vs. MN-ZIF-8	0.5538	0.5013 to 0.6064	Yes	****	<0.0001	A-C
Control vs. GCV@ZIF-8	0.6133	0.5607 to 0.6659	Yes	****	<0.0001	A-D
Control vs. MN/GCV@ZIF-8	0.8191	0.7665 to 0.8716	Yes	****	<0.0001	A-E
MN vs. MN-ZIF-8	0.3321	0.2795 to 0.3846	Yes	****	<0.0001	B-C
MN vs. GCV@ZIF-8	0.3916	0.3390 to 0.4441	Yes	****	<0.0001	B-D
MN vs. MN/GCV@ZIF-8	0.5973	0.5448 to 0.6499	Yes	****	<0.0001	B-E
MN-ZIF-8 vs. GCV@ZIF-8	0.05949	0.006919 to 0.1121	Yes	*	0.0255	C-D
MN-ZIF-8 vs. MN/GCV@ZIF-8	0.2653	0.2127 to 0.3178	Yes	****	<0.0001	C-E
GCV@ZIF-8 vs. MN/GCV@ZIF-8	0.2058	0.1532 to 0.2583	Yes	****	<0.0001	D-E
v-GPCR						
Tukey's Multiple Comparisons Test	Mean Diff.	95.00% CI of Diff.	Below Threshold?	Summary	Adjusted P-Value	
Control vs. MN	0.03883	-0.06047 to 0.1381	No	ns	0.7046	A-B
Control vs. MN-ZIF-8	0.0954	-0.003899 to 0.1947	No	ns	0.0611	A-C
Control vs. GCV@ZIF-8	0.2573	0.1580 to 0.3566	Yes	****	<0.0001	A-D
Control vs. MN/GCV@ZIF-8	0.504	0.4047 to 0.6033	Yes	****	<0.0001	A-E

MN vs. MN-ZIF-8	0.05657	-0.04273 to 0.1559	No	ns	0.3876	B-C
MN vs. GCV@ZIF-8	0.2185	0.1192 to 0.3178	Yes	***	0.0002	B-D
MN vs. MN/GCV@ZIF-8	0.4651	0.3658 to 0.5644	Yes	****	<0.0001	B-E
MN-ZIF-8 vs. GCV@ZIF-8	0.1619	0.06259 to 0.2612	Yes	**	0.0023	C-D
MN-ZIF-8 vs. MN/GCV@ZIF-8	0.4086	0.3093 to 0.5079	Yes	****	<0.0001	C-E
GCV@ZIF-8 vs. MN/GCV@ZIF-8	0.2467	0.1474 to 0.3460	Yes	****	<0.0001	D-E
